# Overexpression of KLF17 Predicts a Favorable Prognosis in Patients with Oral Squamous Cell Carcinoma: A Retrospective Study

**DOI:** 10.3390/medicina56020057

**Published:** 2020-01-30

**Authors:** Yi-Ju Lee, Lung Chan, Chung-Min Yeh, Chien-Hsun Lee, Wen-Wei Sung

**Affiliations:** 1 Department of Pathology, Chung Shan Medical University, Taichung 40201, Taiwan; jasmine.lyl@gmail.com; 2 Department of Pathology, Chung Shan Medical University Hospital, Taichung 40201, Taiwan; 3 School of Medicine, Chung Shan Medical University, Taichung 40201, Taiwan; mrinsect791@gmail.com; 4 Department of Pathology, Changhua Christian Hospital, Changhua 50006, Taiwan; 28935@cch.org.tw; 5 Department of Medical Technology, Jen-Teh Junior College of Medicine, Nursing and Management, Miaoli 35664, Taiwan; 6 Institute of Medicine, Chung Shan Medical University, Taichung 40201, Taiwan; 7 Department of Urology, Chung Shan Medical University Hospital, Taichung 40201, Taiwan

**Keywords:** Krüppel-like factor 17, KLF17, prognosis, oral cancer, oral squamous cell carcinoma, overall survival

## Abstract

*Background and Objectives:* Patients with oral squamous cell carcinoma (OSCC), a common malignancy in Asian countries, have a poor prognosis. We investigated the role of Krüppel-like factor 17 (KLF17) and its prognostic significance in OSCC. *Materials and Methods:* KLF17 expression was measured by immunohistochemical staining of specimens from 283 patients with OSCC. We analyzed correlations between KLF17 expression and clinicopathologic features and between KLF17 expression and overall survival. The prognostic value of KLF17 was tested using Kaplan–Meier analysis and Cox proportional hazard models. *Results:* Among the 283 patients, high KLF17 expression was significantly associated with an early OSCC stage and low *T*-value (*p* = 0.033 and *p* = 0.036, respectively). The five-year survival rates were better in patients with high KLF17 expression than with low expression (66.5% and 49.6%, respectively). The prognostic role of KLF17 was further confirmed through multivariate analysis (hazard ratio 1.506, 95% confidence interval 1.034–2.191, *p* = 0.033). The prognostic value was more significant in patients with a history of betel quid chewing or with a low *T*-value. *Conclusions:* High KLF17 expression can serve as a marker for a favorable prognosis in patients with OSCC. The prognostic role of KLF17 is more significant in patients with a history of betel quid chewing or a low *T*-value.

## 1. Introduction

Oral cancer is one of the most common malignancies in the world. In 2018, an estimated 354,864 new cases of lip and oral cavity cancer were diagnosed worldwide and 177,384 cancer deaths were recorded [1]. According to previous studies, oral squamous cell carcinoma (OSCC) accounts for approximately 90% of oral cancer cases. If OSCC is detected at an early stage (T1), the patient survival rate can be as high as 80%, but if caught at an advanced stage (T3–T4), the survival rate drops to from 20% to 30% [2]. Therefore, the identification of new and specific biomarkers for the early diagnosis of oral cancer is an urgent need [3,4,5,6]. These biomarkers can contribute to clinical decision making and increase survival rates in patients with early-stage oral cancer. 

Krüppel-like factor 17 (KLF17), a new member of the Krüppel-like family of transcription factors, is encoded by the human KLF17 gene, which maps to chromosome 1p34.1 [7,8]. Krüppel-like factors are highly conserved zinc finger transcription factors that serve as key regulators of critical biological cellular processes, including cell proliferation, differentiation, apoptosis, and migration [9,10]. Many studies that have focused on the function of KLF17 in tumorigenesis have reported that KLF17 plays a vital role in cancer development [11,12,13].

Some evidence now supports a relationship between the expression of KLF17 and poor survival in human lung adenocarcinoma, hepatocellular carcinoma, gastric cancer, and papillary thyroid carcinoma [14,15,16,17]. However, the biological role and clinical significance of KLF17 in OSCC are still unclear and might not be significant. In this study, we used immunohistochemical (IHC) staining of microarray sections to investigate the expression of the KLF17 protein in patients with OSCC and to evaluate its clinical and prognostic significance.

## 2. Materials and Methods

### 2.1. Patients

This study included patients with primary OSCC and no other cancer burden. Our study examined 283 tumor samples from patients with OSCC. The cancers were staged according to the AJCC Cancer Staging Manual. The clinicopathological features collected from the established database included risk factors, histological type, differentiation, and TNM stage. The histological diagnoses had been previously confirmed by two pathologists [18]. Patients with missing data or tissue loss during the IHC staining procedure were excluded from the study to reduce the bias from missing data. The study was approved by the Institutional Review Board and Ethics Committee of the Changhua Christian Hospital, Changhua, Taiwan (2014/10/13, IRB No. 131014).

### 2.2. Immunohistochemical Staining of KLF17

The IHC staining was performed at the Department of Surgical Pathology, Changhua Christian Hospital using anti-human KLF17 antibody (abcam-84196; 1:100 dilution), as described previously [18,19]. Immunoreactivity was scored by the pathologists using a previously described scoring protocol [19,20]. The pathologists were blinded to the prognostic data of the study. A final consensus was obtained for each score by having all evaluators view the specimens simultaneously through a multi-headed microscope (Olympus BX51 10-headed microscope). The immunoreactivity scores were defined as cell staining intensity (0–3) multiplied by the percentage of stained cells (0–100%), leading to scores from 0 to 300 [18,19].

### 2.3. Patient and Public Involvement

This study analyzed cancer tissues from a delinked database. Therefore, we did not inform or disseminate information to the patients regarding the research project, outcome investigation, or results. The patients were not involved in the study, including no involvement in the design, recruitment, or conduct of the study. No patient adviser was involved for the contributorship statement.

### 2.4. Statistical Analyses

An χ^2^ test was applied for the continuous or discrete data analysis. The associations between KLF17 expression and overall patient survival were estimated using univariate analysis and the Kaplan–Meier method and assessed further using the log-rank test [20,21]. Data on age, gender, smoking, a history of betel quid chewing, and TNM stage were adjusted for potential confounders using Cox regression models of multivariate analysis, with KLF17 expression fitted as an indicator variable. All statistical analyses were conducted using SPSS statistical software (version 15.0; SPSS Inc., Chicago, IL, USA). All statistical tests were two-sided, and values of *p* < 0.05 were considered statistically significant.

## 3. Results

### 3.1. High KLF17 Expression Levels are More Likely in Early-Stage and Low T-Value Patients with OSCC

**Figure 1:**
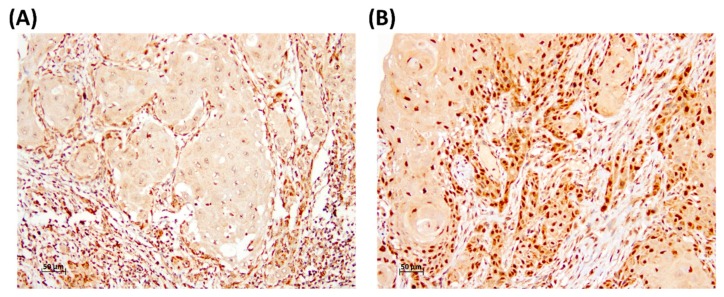
Representative immunostaining of KLF17 in OSCC specimens. Nuclear KLF17 expression levels were (**A**) low and (**B**) high.

We verified the relationships between KLF17 expression and the clinical parameters by recruiting 283 patients with OSCC, and we evaluated KLF17 expression by IHC staining of microarray sections (Figure 1). The scores by the pathologists for the intensity of the KLF17 expression in the nuclei revealed that KLF17 expression was significantly associated with tumor stage and *T*-value. The patients with OSCC with an early stage and a low *T*-value were more likely to have high KLF17 expression (In stage I, 38.3% of patients had low KLF17 and 61.7% had high KLF17, *p* = 0.033; when *T*-value = 1, 40.5% of patients had low KLF17 and 59.5% of patients had high KLF17, *p* = 0.036; Table 1). High expression levels of KLF17 were also more likely in tumors from female than from male patients (high KLF17 expression, 64.4% for females vs. 46.6% for males, *p* = 0.028; Table 1). However, KLF17 expression was not significantly associated with age, smoking, a history of betel quid chewing, tumor differentiation, or N-values.

### 3.2. Patients with OSCC with Advanced Stage or Low KLF17 Expression Had a Poor Clinical Outcome

**Figure 2:**
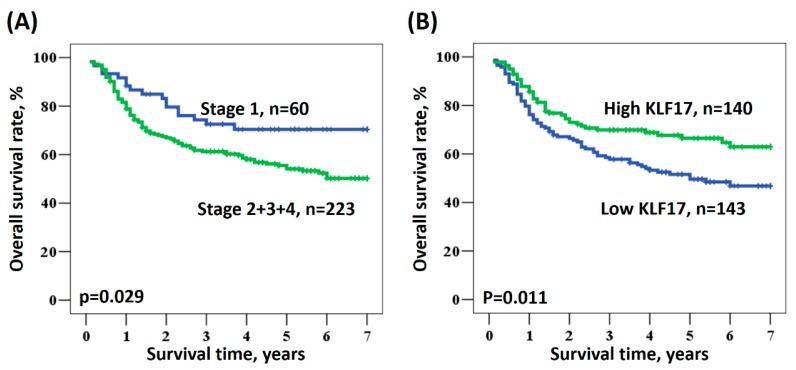
Kaplan–Meier survival curves for patients with oral squamous cell carcinoma (OSCC) according to (**A**) OSCC stage and (**B**) KLF17 expression.

We examined the potential prognostic role of KLF17 expression in patients with OSCC. The survival data were collected from 283 patients, and no data were missing. Kaplan–Meier analysis revealed the relationships between patient survival and different OSCC stages (Figure 2A) or KLF17 expression (Figure 2B). Univariate analysis showed that patients with advanced OSCC stage and low KLF17 expression had a poor survival rate (*p* = 0.029 and 0.011, respectively, Figure 2), indicating that low KLF17 expression and advanced stage were significantly associated with poor clinical outcomes (stage: HR = 1.775, 95% CI = 1.062–2.968, *p* = 0.029; KLF17 expression level: HR = 1.614, 95% CI = 1.114–2.336, *p* = 0.011; Table 2). However, the factors of age, gender, smoking, a history of betel quid chewing, and KLF17 nuclear staining intensity were not significantly associated with prognosis (Table 2).

### 3.3. KLF17 Expression Level as a Prognostic Tool According to Clinicopathological Characteristics of OSCC

The multivariate analysis also revealed a prognostic role for KLF17 in patients with OSCC and different clinicopathological characteristics. Table 3 shows the multivariate analysis of the influence of various parameters on the overall survival of patients with OSCC, adjusted for age, gender, smoking, a history of betel quid chewing, and TNM stage. In our study population, patients with advanced-stage cancer or low KLF17 expression had a significantly poorer prognosis than did patients with early-stage cancer or high KLF17 expression (stage: HR = 1.738, 95% CI = 1.024–2.951, *p* = 0.041; KLF17 expression level: HR = 1.506, 95% CI = 1.034–2.191, *p* = 0.033; Table 3).

Table 4 shows the multivariate analysis of the influence of KLF17 expression on overall survival after adjustment for age, gender, and TNM stage in the patients with OSCC. Low KLF17 expression was significantly associated with poor prognosis in patients with a history of betel quid chewing, low *T*-value, and moderate/poor differentiation (in betel quid chewing patients, HR = 2.934, 95% CI = 1.043–8.249, *p* = 0.041; in low *T*-value patients, HR = 2.471, 95% CI = 1.076–5.673, *p* = 0.033; in moderate/poor differentiation patients: HR = 1.547, 95% CI = 1.035–2.311, *p* = 0.033; Table 4). This is evidence that KLF17 expression could be an independent prognostic marker in patients with low *T*-value tumors, moderately/poorly differentiated tumors, and a history of betel quid chewing, as confirmed by multivariate analysis. This prognostic function of KLF17 was, therefore, significant in patients with specific clinicopathological characteristics.

## 4. Discussion

In this study, we firstly identified that high tumor KLF17 expression is associated with favorable prognosis in patients with OSCC. KLF17 is a member of the Krüppel-like family of transcription factors, which are key regulators of critical biological cellular processes [9,10]. Recent studies have shown that low expression and inactivation of KLF17 can be due to microRNA expression, gene mutations, or the loss of heterozygosity in human tumors, all of which are involved in tumor progression [8,11,22]. Tumors with low KLF17 expression appear to have a higher cell proliferation and metastasis capacity, therefore, patients with these tumors may have a poorer prognosis. By contrast, high KLF17 expression can inhibit tumor growth [12,14]. Therefore, KLF17 can serve as both a predictor of prognosis and a therapeutic target. Decreased KLF17 expression is already an independent prognostic indicator for most human tumors, and low expression is significantly associated with tumor progression. Low KLF17 expression is observed in most human cancers, including colorectal carcinoma, esophageal carcinoma, hepatocellular carcinoma, lung adenocarcinoma, and gastric cancer [12,14,15,16,23,24].

Clinical studies have shown an association between low KLF17 expression and shorter survival time in patients with lung adenocarcinoma and KLF17 expression is significantly associated with tumor stage and size. The overexpression of KLF17 also inhibits the in vitro growth of the A549 and PC-9 lung cancer cell lines, suggesting a potential role for KLF17 in suppressing tumor growth in lung adenocarcinoma [14]. Reduced expression of KLF17 has been strongly correlated with tumor size, pathological N stage, and lymphovascular invasion in gastric cancer and is also an independent predictor of poor survival in patients undergoing gastric cancer surgery [15]. Liu and colleagues reported that low expression of KLF17 in liver cancer is significantly associated with tumor T stage, lymph node stage, M stage, and portal vein tumor thrombosis [16], confirming a tumor-suppressive effect of KLF17 and its potential for use as a prognostic indicator in future treatments.

The clinicopathological characteristics and KLF17 gene expression data provided by the present study for a series of OSCC samples allow the evaluation of a potential role for KLF17 in both clinical treatment and prognosis of OSCC. A key finding of our analysis was that the patients with OSCC whose diagnosis occurred at an earlier stage also had a higher survival rate [2]. This observation prompted us to investigate the prognostic role of KLF17 in our OSCC patient population. Overall, a higher KLF17 expression level was associated with several clinicopathological factors, such as female gender and early tumor stage. Kaplan–Meier analysis confirmed the relationship between patient survival based on different tumor stages and KLF17 expression, as patients with advanced stage disease or low KLF17 expression had poor outcomes (Table 3).

Our multivariate analysis also showed that KLF17 can serve as an independent prognostic marker in patients with low *T*-value, moderate or poor differentiation, and a history of betel quid chewing (Table 4). To the best of our knowledge, this is the first study to indicate that the KLF17 expression level can be an independent prognostic marker for the progression of oral cancer in patients with specific clinicopathological features.

However, our study has some limitations, including the regional source of our cases. Another limitation is that we only investigated overall survival and not relapse-free survival or disease-free survival. Further studies are therefore needed in the future to address these limitations. Nevertheless, our findings do support the likelihood that patients with OSCC tumors with low KLF17 expression have poor clinical outcomes and that KLF17 may be a specific biomarker for evaluating the prognosis of OSCC.

## 5. Conclusions

High KLF17 expression in tumor tissue can serve as a prognostic marker for a favorable survival in patients with OSCC. The prognostic role of KLF17 is more significant in patients with a history of betel quid chewing or with tumors with a low *T*-value.

## Figures and Tables

**Table 1:** Relationships between KLF17 expression and clinical parameters in patients with oral squamous cell carcinoma (OSCC).

Parameters	Case Number	KLF17 Expression		*p-*Value
		Low	High	
Age (year)		56.7 ± 10.7	56.3 ± 12.1	0.782
Gender				
Female	45	16 (35.6)	29 (64.4)	0.028
Male	238	127 (53.4)	111 (46.6)	
Smoking				
No	162	81 (50.0)	81 (50.0)	0.837
Yes	121	62 (51.2)	59 (48.8)	
Betel quid chewing				
No	228	118 (51.8)	110 (48.2)	0.402
Yes	55	25 (45.5)	30 (54.5)	
Differentiation				
Well	40	15 (37.5)	25 (62.5)	0.075
Moderate + Poor	243	128 (52.7)	115 (47.3)	
Stage				
I	60	23 (38.3)	37 (61.7)	0.033
II + III + IV	223	120 (53.8)	103 (46.2)	
*T*-value				
1	79	32 (40.5)	47 (59.5)	0.036
2 + 3 + 4	204	111 (54.4)	93 (45.6)	
*N*-value				
0	173	89 (51.4)	84 (48.6)	0.699
1 + 2 + 3	110	54 (49.1)	56 (50.9)	

**Table 2:** Univariate analysis of the influence of various parameters on the overall survival of patients with oral squamous cell carcinoma (OSCC).

Parameter	Category	Overall Survival			
		5-year Survival (%)	HR	95% CI	*p*-Value
Age	≥57/<57	60.5/57.3	0.992	0.689–1.428	0.966
Gender	Male/Female	56.6/62.6	1.277	0.731–2.232	0.391
Smoking	Yes/No	58.9/56.4	0.919	0.638–1.325	0.653
Betel quid chewing	Yes/No	56.7/59.4	0.819	0.596–1.326	0.417
Stage	II + III + IV/I	54.1/70.4	1.775	1.062–2.968	0.029
KLF17	Low/High	49.6/66.5	1.614	1.114–2.336	0.011

**Table 3:** Multivariate analysis of the influence of various parameters on the overall survival of patients with oral squamous cell carcinoma (OSCC).

Parameter	Category	Overall Survival			
		Mean Survival (years)	HR	95% CI	*p*-Value
Age	≥57/<57	4.7/4.7	0.926	0.637–1.347	0.689
Gender	Male/Female	4.6/5.0	1.171	0.651–2.106	0.598
Smoking	Yes/No	4.8/4.6	0.894	0.580–1.378	0.612
Betel quid chewing	Yes/No	5.0/4.6	0.826	0.470–1.450	0.505
Stage	II + III + IV/I	4.5/5.4	1.738	1.024–2.951	0.041
KLF17	Low/High	4.3/5.1	1.506	1.034–2.191	0.033

**Table 4:** Multivariate analysis of the influence of KLF17 expression according to clinical parameters on overall survival in patients with oral squamous cell carcinoma (OSCC).

Parameter	Overall Survival ^1^			
	5-year Survival (%) ^2^	HR	95% CI	*p*-Value
All cases ^2^	49.6/66.5	1.506	1.034–2.191	0.033
Age (year)				
<57	50.3/64.8	1.351	0.828–2.206	0.229
≥57	49.2/69.0	1.619	0.896–2.926	0.110
Gender				
Female	50.6/72.2	1.512	0.520–4.394	0.447
Male	49.4/65.5	1.475	0.987–2.203	0.058
Smoking				
No	49.0/65.1	1.341	0.817–2.201	0.245
Yes	50.4/68.2	1.701	0.951–3.041	0.073
Betel quid chewing				
No	52.0/62.3	1.258	0.839–1.888	0.267
Yes ^2^	38.2/79.4	2.934	1.043–8.249	0.041
Differentiation				
Well	64.6/76.2	1.151	0.267–4.967	0.851
Moderate + Poor ^3^	47.9/64.2	1.547	1.035–2.311	0.033
Stage				
I	60.6/77.4	1.397	0.522–3.736	0.506
II + III + IV	47.5/62.5	1.440	0.958–2.164	0.080
*T*-value				
1	52.9/78.1	2.471	1.076–5.673	0.033
2 + 3 + 4	48.5/60.4	1.307	0.856–1.995	0.214
*N*-value				
0	61.6/75.9	1.652	0.933–2.926	0.085
1 + 2 + 3	29.8/51.3	1.589	0.952–2.652	0.076

^1^ Adjusted for age, gender, and stage; ^2^ Adjusted stage: HR = 41.078, 95% CI = 1.984–850.721, *p* = 0.016; ^3^ Adjusted stage: HR = 1.434, 95% CI = 0.841–2.445, *p* = 0.186.

## References

[1] Bray F., Ferlay J., Soerjomataram I., Siegel R.L., Torre L.A., Jemal A. (2018). Global cancer statistics 2018: GLOBOCAN estimates of incidence and mortality worldwide for 36 cancers in 185 countries. CA Cancer J. Clin..

[2] Dumache R., Rogobete A.F., Andreescu N., Puiu M. (2015). Genetic and Epigenetic Biomarkers of Molecular Alterations in Oral Carcinogenesis. Clin. Lab..

[3] Ferlazzo N., Curro M., Zinellu A., Caccamo D., Isola G., Ventura V., Carru C., Matarese G., Ientile R. (2017). Influence of MTHFR Genetic Background on p16 and MGMT Methylation in Oral Squamous Cell Cancer. Int. J. Mol. Sci..

[4] Kale A.D., Angadi P.V. (2019). Tumor budding is a potential histopathological marker in the prognosis of oral squamous cell carcinoma: Current status and future prospects. J. Oral Maxillofac. Pathol..

[5] Matarese G., Curro M., Isola G., Caccamo D., Vecchio M., Giunta M.L., Ramaglia L., Cordasco G., Williams R.C., Ientile R. (2015). Transglutaminase 2 up-regulation is associated with RANKL/OPG pathway in cultured HPDL cells and THP-1-differentiated macrophages. Amino Acids.

[6] Zhang L., Meng X., Zhu X.W., Yang D.C., Chen R., Jiang Y., Xu T. (2019). Long non-coding RNAs in Oral squamous cell carcinoma: Biologic function, mechanisms and clinical implications. Mol. Cancer.

[7] McConnell B.B., Yang V.W. (2010). Mammalian Kruppel-like factors in health and diseases. Physiol. Rev..

[8] van Vliet J., Crofts L.A., Quinlan K.G., Czolij R., Perkins A.C., Crossley M. (2006). Human KLF17 is a new member of the Sp/KLF family of transcription factors. Genomics.

[9] Limame R., Op de Beeck K., Lardon F., De Wever O., Pauwels P. (2014). Kruppel-like factors in cancer progression: Three fingers on the steering wheel. Oncotarget.

[10] Tetreault M.P., Yang Y., Katz J.P. (2013). Kruppel-like factors in cancer. Nat. Rev. Cancer.

[11] Zhou S., Tang X., Tang F. (2016). Kruppel-like factor 17, a novel tumor suppressor: Its low expression is involved in cancer metastasis. Tumour Biol..

[12] Li S., Qin X., Cui A., Wu W., Ren L., Wang X. (2015). Low expression of KLF17 is associated with tumor invasion in esophageal carcinoma. Int. J. Clin. Exp. Pathol..

[13] Ali A., Bhatti M.Z., Shah A.S., Duong H.Q., Alkreathy H.M., Mohammad S.F., Khan R.A., Ahmad A. (2015). Tumor-suppressive p53 Signaling Empowers Metastatic Inhibitor KLF17-dependent Transcription to Overcome Tumorigenesis in Non-small Cell Lung Cancer. J. Biol. Chem..

[14] Cai X.D., Zhou Y.B., Huang L.X., Zeng Q.L., Zhang L.J., Wang Q.Q., Li S.L., Feng J.Q., Han A.J. (2012). Reduced expression of Kruppel-like factor 17 is related to tumor growth and poor prognosis in lung adenocarcinoma. Biochem. Biophys. Res. Commun..

[15] Peng J.J., Wu B., Xiao X.B., Shao Y.S., Feng Y., Yin M.X. (2014). Reduced Kruppel-like factor 17 (KLF17) expression correlates with poor survival in patients with gastric cancer. Arch. Med. Res..

[16] Liu F.Y., Deng Y.L., Li Y., Zeng D., Zhou Z.Z., Tian D.A., Liu M. (2013). Down-regulated KLF17 expression is associated with tumor invasion and poor prognosis in hepatocellular carcinoma. Med. Oncol..

[17] Ye W.C., Gao L., Huang J., Fang X.M., Xie G. (2014). Suppressed Kruppellike factor 17 expression induces tumor proliferation, metastasis and a poor prognosis in papillary thyroid carcinoma. Mol. Med. Rep..

[18] Hwang J.C., Sung W.W., Tu H.P., Hsieh K.C., Yeh C.M., Chen C.J., Tai H.C., Hsu C.T., Shieh G.S., Chang J.G. (2015). The Overexpression of FEN1 and RAD54B May Act as Independent Prognostic Factors of Lung Adenocarcinoma. PLoS ONE.

[19] Sung W.W., Lin Y.M., Wu P.R., Yen H.H., Lai H.W., Su T.C., Huang R.H., Wen C.K., Chen C.Y., Chen C.J. (2014). High nuclear/cytoplasmic ratio of Cdk1 expression predicts poor prognosis in colorectal cancer patients. BMC Cancer.

[20] Sung W.W., Wang Y.C., Cheng Y.W., Lee M.C., Yeh K.T., Wang L., Wang J., Chen C.Y., Lee H. (2011). A polymorphic -844T/C in FasL promoter predicts survival and relapse in non-small cell lung cancer. Clin. Cancer Res..

[21] Sung W.W., Wang Y.C., Lin P.L., Cheng Y.W., Chen C.Y., Wu T.C., Lee H. (2013). IL-10 promotes tumor aggressiveness via upregulation of CIP2A transcription in lung adenocarcinoma. Clin. Cancer Res. Off. J. Am. Assoc. Cancer Res..

[22] Camacho-Vanegas O., Narla G., Teixeira M.S., DiFeo A., Misra A., Singh G., Chan A.M., Friedman S.L., Feuerstein B.G., Martignetti J.A. (2007). Functional inactivation of the KLF6 tumor suppressor gene by loss of heterozygosity and increased alternative splicing in glioblastoma. Int. J. Cancer.

[23] Gao H.L., Zhou N., Sun Z., Dou X.L., Guan M., Bai C.M. (2016). KLF17 Expression in Colorectal Carcinoma and Its Clinical Significance. Zhongguo Yi Xue Ke Xue Yuan Xue Bao.

[24] Lu X.J., Shi Y., Chen J.L., Ma S. (2015). Kruppel-like factors in hepatocellular carcinoma. Tumour Biol..

